# Effect of a dietary nutraceutical “STRUCTURE-Joint” on response of horses to intra-articular challenge with IL-1: implications for tissue adaptation to stress

**DOI:** 10.1093/tas/txae172

**Published:** 2024-12-07

**Authors:** Lindsay Korac, Nadia Golestani, Jennifer MacNicol, Jamie Souccar-Young, Sophie Witherspoon, Arayih Wildish, Sydney Topfer, Wendy Pearson

**Affiliations:** Department of Animal Biosciences, University of Guelph, Guelph, ON N1G 2W1, Canada; Department of Animal Biosciences, University of Guelph, Guelph, ON N1G 2W1, Canada; Department of Animal Biosciences, University of Guelph, Guelph, ON N1G 2W1, Canada; Department of Animal Biosciences, University of Guelph, Guelph, ON N1G 2W1, Canada; Department of Animal Biosciences, University of Guelph, Guelph, ON N1G 2W1, Canada; Department of Animal Biosciences, University of Guelph, Guelph, ON N1G 2W1, Canada; Department of Animal Biosciences, University of Guelph, Guelph, ON N1G 2W1, Canada; Department of Animal Biosciences, University of Guelph, Guelph, ON N1G 2W1, Canada

**Keywords:** equine physiology, exercise performance, inflammation modulation, nutraceuticals, polyunsaturated fatty acids

## Abstract

The purpose was to determine local (articular) and systemic effects of intra-articular interleukin-1 in horses supplemented with a dietary PUFA supplement [STRUCTURE-Joint (**ST-J**)]. Sixteen (16) healthy, mature, light breed horses were randomly assigned to diets containing 0 or 120 mL (*n* = 8 per group) of ST-J for 30 d. On days 0 (prior to beginning supplementation) and 27, recombinant equine interleukin-1β (**reIL-1 β**) (75 ng) was injected into the left or right intercarpal joint to induce mild, transient synovitis. Synovial fluid was obtained by aseptic arthrocentesis at postinjection hour 0 (immediately prior to IL-1 injection), 6, 12, and 72. ST-J supplementation for 30 d significantly increased synovial fluid nitric oxide, and resolvin D1 compared with the unsupplemented control group and significantly increased PGE_2_ levels and reduced joint circumference in the ST-J treated horses on day 30 compared to the same group of horses on day 0. There was also a significant increase in plasma hemoglobin, free and total bilirubin, and decrease in plasma glucose. These data provide evidence for the usefulness of ST-J to modulate physiological variables with importance in exercise performance and tissue adaptation to exercise stress and further research on this product is warranted.

## Teaser

This study demonstrates that the dietary supplement STRUCTURE-Joint (**ST-J** ) supports adaptation to exercise stress in horses by improving inflammatory responses and increasing key mediators of joint metabolism. These findings highlight the potential of ST-J to benefit equine performance.

## INTRODUCTION

Polyunsaturated fatty acid (**PUFA**) supplementation in horses is a common dietary strategy for improving joint (Bradbery et al., 2018), skin and coat (O’Neill et al., 2002), respiratory (Nogradi et al, 2015), gastrointestinal (Pagan et al., 2022), and reproductive health (Bazzano et al., 2021; Ferreira et al., 2021; Catandi et al., 2022), as well as for improving adaptation to exercise in muscle (Mrugala et al., 2021) and blood (O’Connor et al., 2004; Hess et al., 2019). While evidence for the potential benefit of feeding PUFA supplements to horses appears robust, the majority of commercially available equine PUFA supplements are marketed in the absence of scientific evidence for efficacy and instead rely on indirect marketing with all its associated risks and “scientific mistruths” (McIlwraith, 2004).

STRUCTURE-Joint (**ST-J**; EC Nutraceuticals, Erin ON Canada) is a PUFA supplement formulated to increase provision Ω6 to Ω3 fatty acids in the equine diet. Both Ω6 and Ω3 fatty acids are essential fatty acids, meaning that the horse cannot manufacture them, and they must therefore be provided in the diet. Generally speaking, the Ω6 fatty acids (notably, linoleic acid, arachidonic acid, and gamma-linolenic acid) promote tissue response to inflammatory stimuli (Burron et al., 2024), whilst the Ω3 fatty acids (including ɑ-linolenic acid, eicosapentaenoic acid, and docosahexaenoic acid) promote *resolution* of response to inflammatory stimuli (Oakes et al., 2024). Both of these actions are critical to effective and dynamic tissue responses to stressful stimuli; thus it is increasingly understood that provision of Ω6 to Ω3 fatty acids encourages appropriate inflammatory responses followed by rapid and efficient resolution of inflammation (Burron et al., 2024). This transient cycle of inflammation followed by inflammation resolution is absolutely essential to tissue adaptation to repetitive stressful stimuli such as exercise (Langston et al., 2024). To the authors’ knowledge, there are no studies that define an “optimum” ratio of Ω6 to Ω3 fatty acids in equine diets. But it is known that fresh forages (i.e., pasture), representing what is arguably the “natural” diet of the horse, have a ratio of around 0.3:1 (Burron et al., 2024). This is in stark contrast to common horse feed ingredients, including oats (19.5:1), corn (55:1), barley (9.1:1), and brewer’s grains (9.3:1), which have very high Ω6 to Ω3 ratios (FeedXL, 2024). Thus, feed supplements that promote lower Ω6 to Ω3 ratios can be expected to improve the dynamic inflammatory responses to exercise stress.

For the current study, we tested the hypothesis that providing a dietary PUFA supplement with a low Ω6 to Ω3 ratio to horses promotes transient inflammatory response to a stimulus, which is limited by formation of pro-resolving compounds. The objective was to evaluate the local (articular) and systemic response of horses to a mild intra-articular challenge with recombinant equine interleukin 1β (**IL-1**) after 30 d of consuming ST-J.

## MATERIALS AND METHODS

Use of horses was reviewed and approved by the University of Guelph Animal Care Committee (AUP # 4764), in accordance with the guidelines of the Canadian Council on Animal Care.

All chemicals and reagents were purchased from Sigma Aldrich (Mississauga, ON) unless otherwise indicated. Absorbances for all assays were obtained from a Victor 1420 Microplate Reader (Perkin Elmer, Woodbridge, ON). All biological samples (blood and synovial fluid) were placed on ice immediately after collection and remained there until processing, as described below.

### Horses

Sixteen healthy, mixed breed, mature horses (Table 1) without a history of articular disease from the OMAFRA Arkell Equine Research Station (Arkell ON) were recruited into the randomized, controlled study. They were group-housed in an open turnout area with unrestricted access to a loafing barn bedded with straw. Horses had access to *ad lib* hay, trace mineral salt, and water and had been adapted to their diet and environment for at least 12 mo prior to inclusion in the study. No additional feed was provided to any of the horses at any time. The hay had a total fatty acid content of 0.75% DM. Table 2 provides the relative fatty acid composition. Horses were blocked according to age and body weight (see Table 1), and then randomly assigned to receive ST-J (Table 3) at a dose rate 120 mL (ST-J; *n* = 8) of oil by oral syringe once per day. Untreated control (**CON**; *n* = 8) horses did not receive any additional supplement.

**Table 1.** Horse demographics

Name	Treatment	Age (yr)	Weight (kg)	Sex
Samson	CON	19	630	Gelding
Olivia	CON	7	530	Mare
Janie	CON	18	590	Mare
Mia	CON	5	400	Mare
Missy	CON	15	485	Mare
Amelia	CON	4	485	Mare
Ava	CON	14	500	Mare
Snickers	CON	5	485	Mare
*Mean (±SEM)*		10.9 (±2.22) yr	513.1 (±25.1) kg	
Diosa	ST-J	10	500	Mare
Isabella	ST-J	10	590	Mare
Twix	ST-J	4	380	Mare
Melrose	ST-J	13	500	Mare
PNut	ST-J	26	490	Gelding
Coral	ST-J	14	510	Mare
Sophia	ST-J	3	460	Mare
Maggie	ST-J	22	510	Mare
*Mean (±SEM)*		12.8 (±2.8) yr	492.5 (±20.7) kg	

**Table 2.** Fatty acid composition of hay (dry matter basis)

Total fatty acid (%)	0.72
C 16:0 Palmitic acid (%TFA)	25.00
C 18:0 Stearic acid (%TFA)	4.17
C 18:1 Oleic acid (%TFA)	8.33
C 18:2 Linoleic acid (%TFA)	9.72
C 18:3 Linolenic acid (%TFA)	51.29

**Table 3.** Fatty acid analysis of ST-J (per 100 g)

Fat	95.2 g
Free fatty acids	1.03 %
Calories	876
Total vitamin E (tocopherols)	2110 IU
Total omega-3 isomers	11.3 g
Total omega-6 isomers	44.8 g
Omega 6:3 ratio	3.96:1
C22:6 Docosahexaenoic acid (DHA)	0.999 g
C20:5 Eicosapentaenoic acid (EPA)	1.54 g

### Synovitis Induction and Synovial Fluid Collection

On days 0 and 27, the hair over the intercarpal joint was clipped, and the skin surface was desensitized with liberal application of topical lidocaine/prilocaine cream (Emla cream: Astra Pharmaceutical). After 15 min of emla contact time, the site was aseptically prepared with 70% isopropyl alcohol and 4% chlorhexadine. Subsequently, a 22-gauge 1″ sterile needle was inserted into the intercarpal joint, and an empty, sterile 3 mL syringe was attached to the hub. Approximately 1.5 mL of synovial fluid was aspirated into the syringe. The syringe was then removed, and the synovial fluid sample was immediately injected into a 5 mL vacutainer containing heparin. A second 3 mL syringe containing 75 μg of IL-1 in 500 μL sterile saline was then connected to the inserted needle hub, and the reIL-1 β solution was slowly injected. The needle was then removed. Additional synovial fluid samples were obtained at 6, 12, and 72 h postinjection hour (**PIH**). These timepoints were selected to capture the peak in the primary outcome measure (synovial fluid prostaglandin E_2_ [**PGE**_**2**_]) following intra-articular reIL-1 β challenge (Pearson et al., 2009). day 30 represents the 72 h sample from the day 27 reIL-1 β injection.

Synovial fluid samples were placed on ice as soon as they were collected and remained there until samples were obtained from all horses (maximum 5 h). They were then transported to the lab, centrifuged (5,000 × g for 15 min), and the supernatant was placed into a 1.5 mL Eppendorf tube containing 10 μg of indomethacin. Synovial fluid samples were then stored at −80 °C until analysis for PGE_2_ (multispecies ELISA; Arbor Assays, Mississauga ON, CAT# K051-H5), glycosaminoglycan (**GAG**; dimethyl methylene blue assay; MacNicol et al., 2018), resolvin D1 (**RvD1**; equine ELISA; AffiGen Life Science Supply Chain, Bailey’s Harbor WI; CAT# AFG-SB-7816), nitric oxide (**NO**; Griess Reaction; MacNicol et al., 2018), and chondroitin sulfate epitope 846 (**CS846**; ELISA; IBEX, Montreal QC; CAT# 60-1004).

Prior to analysis, all synovial fluid samples were thawed and nutated for 1 h with a hyaluronidase solution (4 mg/mL; HAse) (Jayadev et al., 2012) at a 1:1 dilution. Diluted samples were then centrifuged at 1,000 × *g* for 5 min, and the supernatant was removed and used in the assays.

### Clinical Outcomes

Clinical outcomes [lameness (AAEP Lameness Scale—Table 4)], joint circumference (flexible measuring tape around the intercarpal joint from a standing, loaded position), and rectal temperature (rectal thermometer) were obtained immediately prior to arthrocentesis at 0, 6, 12 and 72 PIH. Lameness exam was conducted on a concrete surface by a licenced veterinary practitioner (Dr Amanda Jowett). Each horse was first walked, then trotted in-hand by the same handler in a straight line away from and then back towards the veterinarian. The IL-1-injected joint was then held in flexion for 60 s after which time the horse was immediately trotted in-hand away from the veterinarian.

**Table 4.** AAEP Lameness grading system

Score	
0	Lameness not perceptible under any circumstances
1	Lameness is difficult to observe, is not consistently apparent
2	Lameness difficult to observe at a walk or when trotting in a straight line but is consistently apparent under flexion
3	Lameness consistently observable at a trot under all circumstances
4	Lameness obvious at a walk
5	Lameness produces minimal weight bearing in motion and/or at rest or a complete inability to move

### Systemic (Blood) Outcomes

On days 0 and 27, jugular venous blood was collected by venipuncture into silicone-coated and EDTA-coated vacutainers. Samples were sent to the Animal Health Laboratory at the University of Guelph within 5 h of collection for determination of Complete Blood Count (**CBC**) [white blood cell count (**WBC)**, red blood cell count (**RBC**), hemoglobin (**Hb**), hematocrit (**HCT**), mean corpuscular volume (**MCV**), mean hemoglobin content (**MHC**), mean corpuscular hemoglobin concentration (**MCHC**), red cell distribution width (**RDW**), platelets, mean plasma volume (**MPV**), total solid protein (**T.S. protein**), segmented neutrophils (**Seg neutrophils**), lymphocytes, monocytes, eosinophils, basophils, rouleux] and Equine Serum Profile [calcium, phosphorus, magnesium, sodium (**Na**), potassium (**K**), chloride, carbon dioxide, anion gap, NaK ratio, total protein, albumin (**A**), globulin (**G**), AG ratio, urea, creatinine, glucose, cholesterol, bilirubin, conjugated bilirubin, alkaline phosphatase, gamma glutamyl transferase (**GGT**), aspartate aminotransferase (**AST**), glutamate dehydrogenase (**GLDH**), calculated osmolality, serum amyloid A]. These analyses were conducted to identify systemic immune or other systemic effects (including potential adverse effects) of treatment.

### Statistical Methods

All data are reported as mean ± SEM unless otherwise indicated. Data were analyzed using a 3-way repeated measures ANOVA with respect to dietary treatment, PIH, and Sample Day. Two-way ANOVA with respect to dietary treatment and PIH for each sample day, and with respect to PIH and Sample Day for each dietary treatment were also conducted. When a significant *F*-ratio was obtained, the Tukey’s post hoc test was used to identify significantly different means. Trends were identified when 0.1 > *P* > 0.05. Significance was accepted when *P* ≤ 0.05.

### Clinical Outcomes

#### Lameness grade.

Three-way ANOVA detected no significant effect of reIL-1 β on trot lameness score (*P* = 0.22). Overall, trot lameness score was significantly higher on day 0 (0.8/5) than on day 30 (0.5/5) (*P* = 0.005), and ST-J horses had a significantly lower lameness score than CON horses (*P* < 0.001).

Two-way analysis detected a significant decrease in lameness score in CON horses between days 0 (1.2/5) and 30 (0.7/5) (*P* = 0.05). Lameness score in ST-J horses also significantly decreased between days 0 (0.5/5) and 30 (0.2/5) (*P* = 0.04).

ST-J horses had significantly lower lameness score than CON horses on days 0 (p = 0.003) and 30 (*P* = 0.002).

#### Rectal temperature.

**Figure 1.**
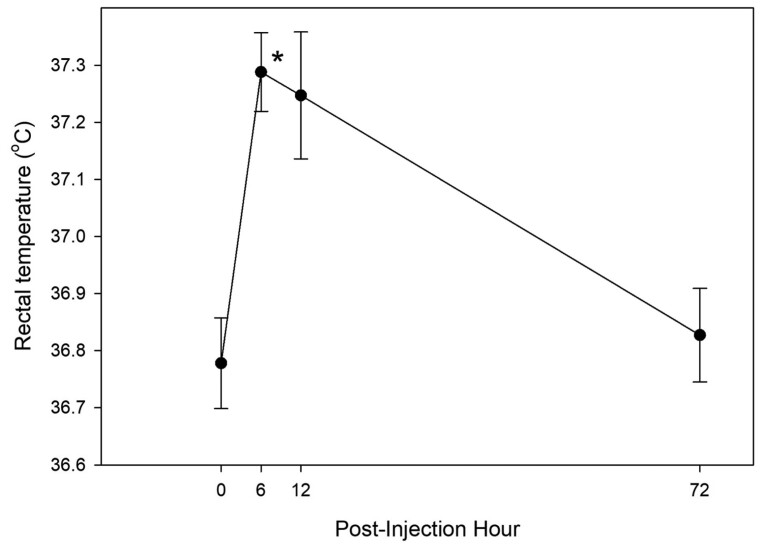
Rectal temperature (°C) following intra-articular injection of IL-1 into one intercarpal joint of healthy horses (*n* = 16). * denotes significant change from baseline.

Three-way ANOVA detected a significant increase in rectal temperature at PIH 6 (*P* < 0.001) (Figure 1).

Two-way analysis shows a significant increase in rectal temperature CON (*P* < 0.001) and ST-J (*P* = 0.01) at PIH 6, and there were no differences between days 0 and 30 for either group.

#### Joint circumference.

Three-way ANOVA detected no significant effect of reIL-1 β injection on joint circumference (*P* = 0.26). Overall, ST-J horses had significantly higher joint circumference than CON horses (*P* < 0.001). There was a tendency (*P* = 0.08) for joint circumference to be lower on day 30 than day 0 in all horses.

Two-way analysis shows no effect of reIL-1 β or day on joint circumference in CON horses. Joint circumference in ST-J horses was significantly higher than in CON horses on day 0 (*P* < 0.001) and day 30 (*P* = 0.002). On day 0, joint circumference in ST-J horses was significantly higher than CON horses at PIH 6 (*P* = 0.008), but on day 30 there were no differences between groups at any time point.

**Figure 2.**
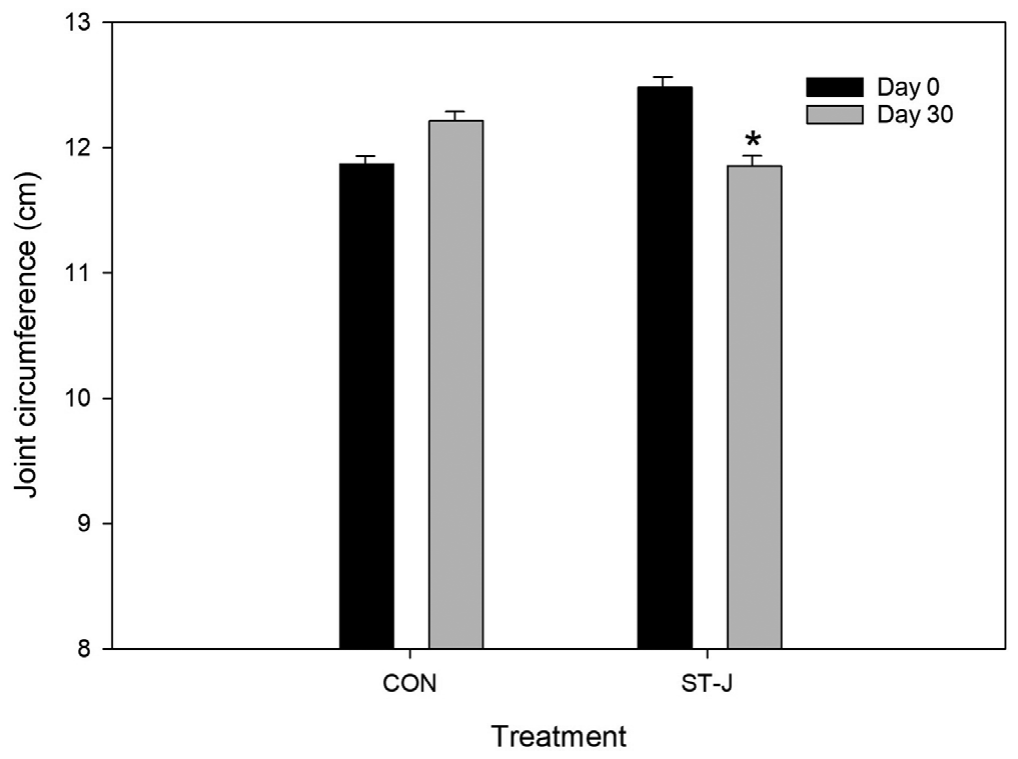
Mean joint circumference (cm) of intercarpal joints injected with IL-1 of horses receiving an unsupplemented CON diet (*n* = 8) or a diet containing 120 mL per day of ST-J (*n* = 8) at baseline (day 0, prior to supplementation) and then after 30 d. *denotes significant difference between days 0 and 30 within a single treatment group (*P* < 0.05).

In ST-J horses, joint circumference was significantly lower on day 30 (12.1 ± 0.07 cm) than day 0 (12.5 ± 0.08 cm) (*P* = 0.02) (Figure 2).

### Synovial Fluid Outcomes

#### Prostaglandin E2.

**Figure 3.**
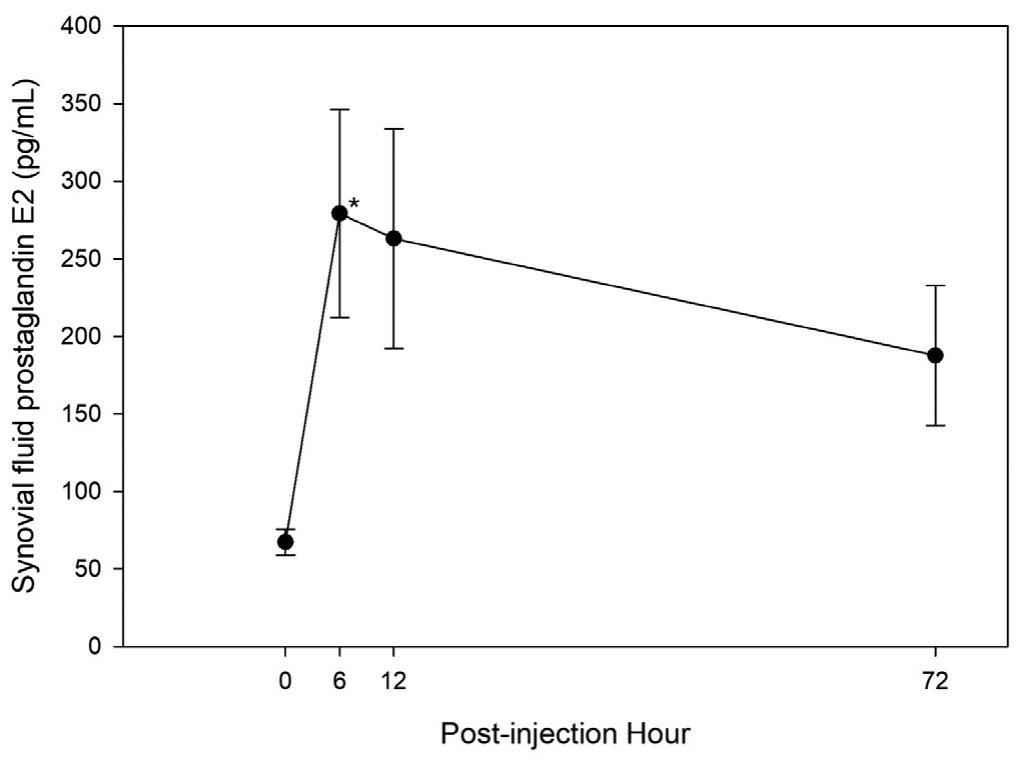
Mean synovial fluid PGE_2_ from all horses on days 0 and 30 at 0, 6, 12, and 72 h after injection of IL-1 in healthy horses (*n* = 16). *denotes significant change from baseline (*P* < 0.05).

Three-way ANOVA detected a significant increase in synovial fluid PGE_2_ at PIH 6 (*P* = 0.02) (Figure 3). Overall, synovial fluid PGE_2_ was significantly lower on day 0 than day 30 (*P* = 0.02), and there was no significant difference between CON and ST-J horses (*P* = 0.22).

**Figure 4.**
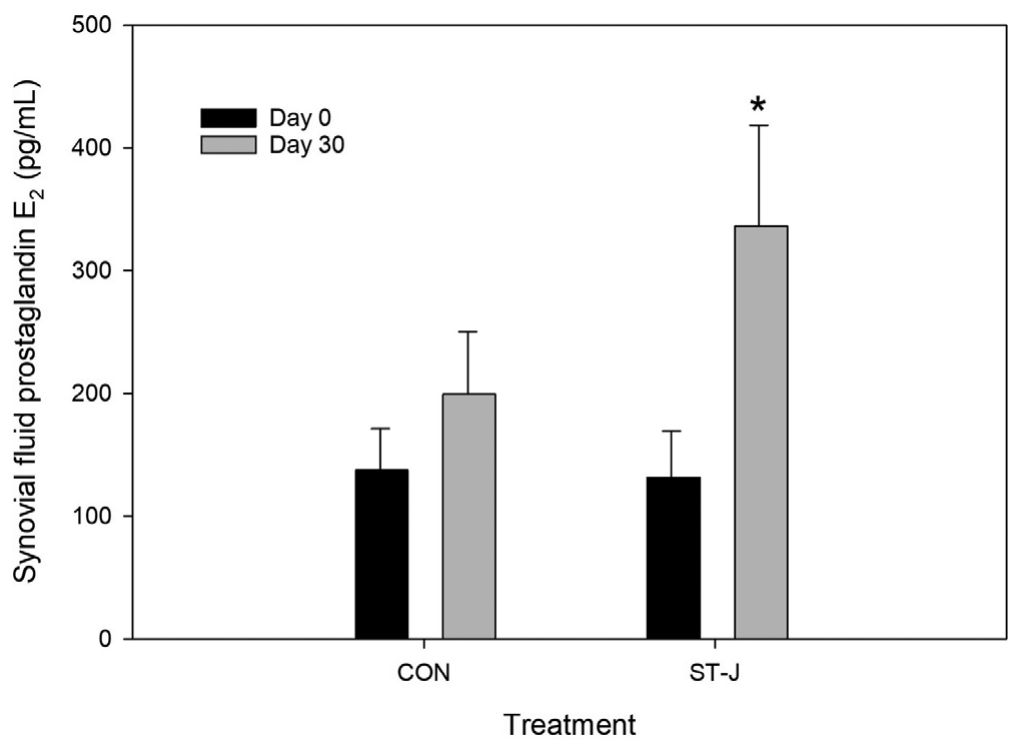
Mean synovial fluid PGE_2_ (pg/mL) in horses receiving an unsupplemented CON diet (*n* = 8) or a diet containing 120 mL per day of ST-J (*n* = 8) at baseline (day 0, prior to supplementation) and then after 30 d. * denotes significant difference between days 0 and 30 within a single treatment group (*P* < 0.05).

Two-way ANOVA detected no significant differences in synovial fluid PGE_2_ between CON and ST-J on days 0 (*P* = 0.96) or 30 (*P* = 0.17). There were no significant differences in synovial fluid PGE_2_ between days 0 and 30 in CON horses (*P* = 0.28). Synovial fluid PGE_2_ was significantly lower on day 0 than day 30 in ST-J horses (*P* = 0.04) (Figure 4).

#### Nitric oxide.

Three-way ANOVA detected no significant effect of reIL-1 β on synovial fluid NO (*P* = 0.83). There were no significant effects of treatment (*P* = 0.64) or day (0.11) on synovial fluid NO.

**Figure 5.**
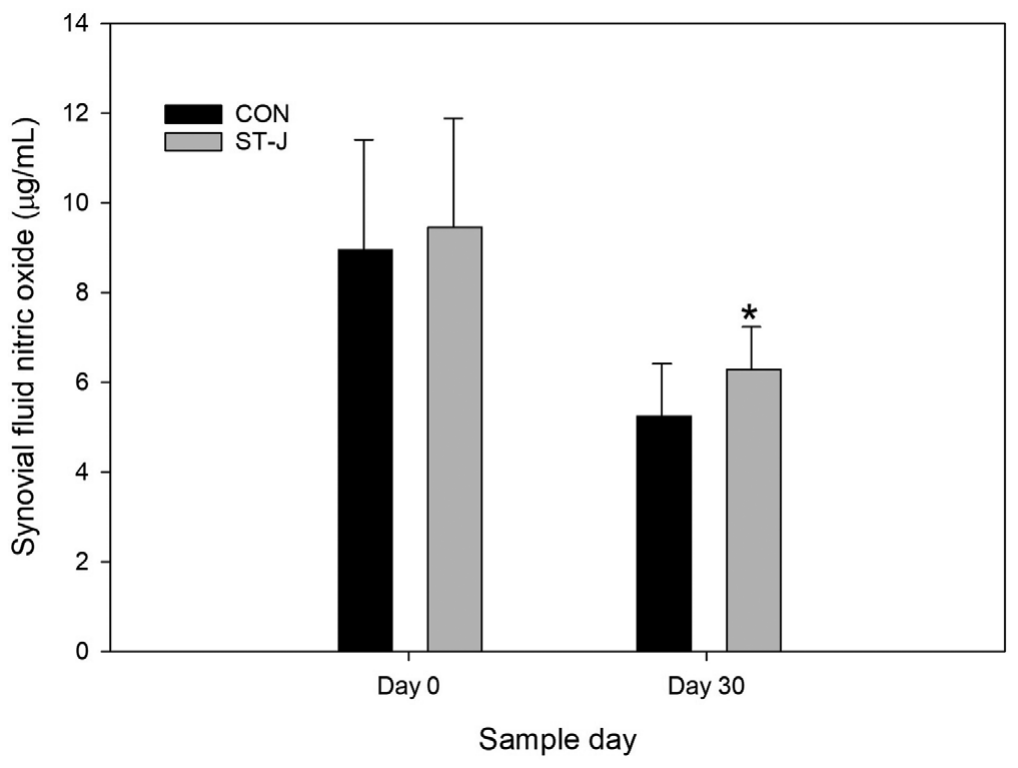
Mean synovial fluid nitric oxide (μg/mL) at baseline (day 0, prior to supplementation) and then 30 d after an unsupplemented CON diet (*n* = 8) or a diet containing 120 mL per day of ST-J (*n* = 8). * denotes significant difference between groups on a single day (*P* < 0.05).

Two-way ANOVA detected no significant difference between days 0 and 30 in synovial fluid NO in CON horses (*P* = 0.24). There were also no differences in synovial fluid NO between days 0 and 30 in ST-J horses (0.28). On day 30, ST-J horses had significantly higher NO concentration than CON horses (*P* = 0.04) (Figure 5). There were no differences between groups on day 0 (*P* = 0.84).

#### Glycosaminoglycan.

Three-way ANOVA detected no significant effect of reIL-1 β on synovial fluid GAG (*P* = 0.19). There were no significant effects of Day (*P* = 0.25) or treatment (*P* = 0.59) on synovial fluid GAG. Synovial fluid GAG was not different between days 0 and 30 in CON (*P* = 0.57) or ST-J horses (*P* = 0.23). There were no differences in synovial fluid GAG concentrations between CON and ST-J horses on day 0 (*P* = 0.58) or 30 (*P* = 0.83).

#### Synovial fluid chondroitin sulphate epitope 846.

Three-way ANOVA detected no significant effect of reIL-1 β on synovial fluid CS846 (*P* = 0.42). Day was also not a significant contributor to changes in CS846 (*P* = 0.32). Overall, horses in the ST-J group had significantly lower CS846 than CON horses (*P* = 0.008).

**Figure 6.**
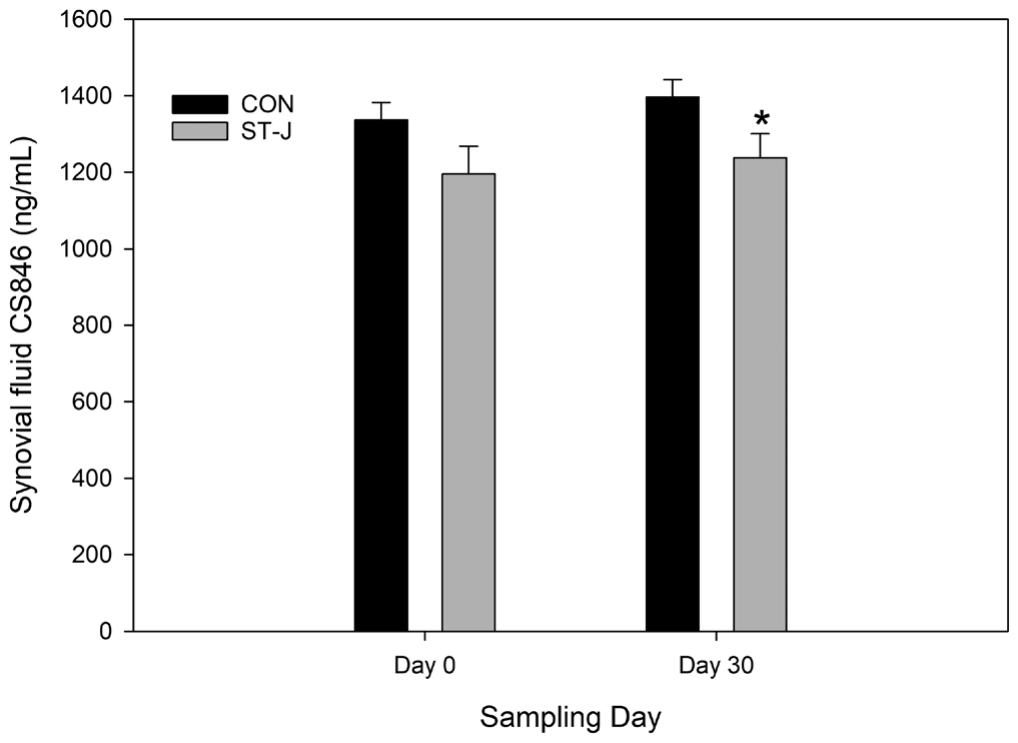
Mean synovial fluid nitric CS846 (ng/mL) at baseline (day 0, prior to supplementation) and then 30 d after an unsupplemented CON diet (*n* = 8) or a diet containing 120 mL per day of ST-J (*n* = 8). * denotes significant difference between groups on the same day (*P* < 0.05).

Two-way ANOVA detected no significant difference between days 0 and 30 in synovial fluid CS846 in CON horses (*P* = 0.38) or ST-J horses (*P* = 0.56). CS846 concentration tended to be lower in ST-J horses than CON horses on day 0 (*P* = 0.07) and day 30 (*P* = 0.05) (Figure 6).

#### Resolvin D1.

Three-way ANOVA detected no significant effect of reIL-1 β on synovial fluid RvD1 (*P* = 0.32). Overall, there was a tendency for ST-J horses to have higher RvD1 than CON horses (*P* = 0.06). There was also a tendency for RvD1 to be higher on day 30 than day 0 (*P* = 0.1).

**Figure 7.**
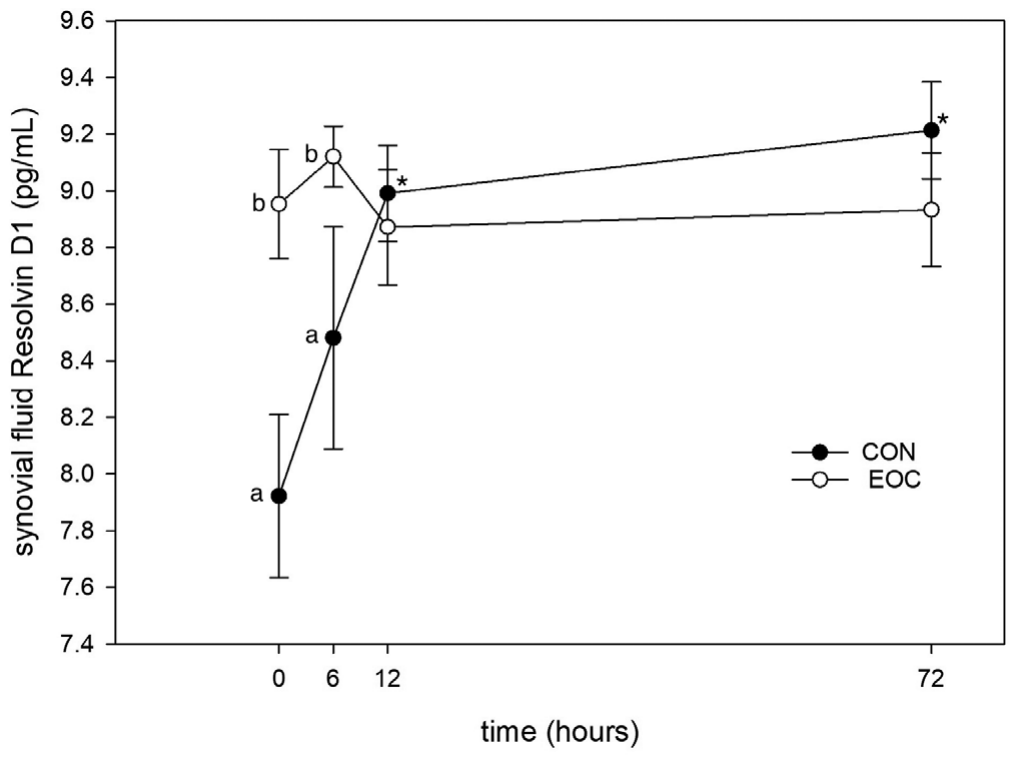
Synovial fluid RvD1 following injection of IL-1 in healthy horses (*n* = 8 per group) after 30 d of supplementation with 120 mL ST-J or an unsupplemented CON diet. * denotes significant difference from baseline within a single group; lower-case letters denote differences between groups at a single time point.

Two-way ANOVA detected no significant difference between ST-J and CON horses at day 0 (*P* = 0.36). However, on day 30 ST-J horses had significantly higher RvD1 at PIH 0 (*P* = 0.02) and 6 (*P* = 0.02) compared with CON horses. Day 30 also saw a significant increase in RvD1 at PIH 12 (*P* = 0.005) and 72 (*P* = 0.002) (Figure 7) in CON horses.

### Systemic (Blood) Outcomes

All blood parameters remained within normal reference intervals throughout the trial in both treatment groups.

#### Complete blood count.

CBC data for days 0 and 30 are provided in Table 5. There were no statistically significant effects of dietary treatment or day in any CBC outcomes.

**Table 5.** CBC outcome measures

Outcome measure	Dietary treatment	Day 0	Day 30	*P*-value
				(Day 0 × Day 30)
WBC	C^1^	6.537 ± 1.31	7.35 ± 1.33	0.90
	ST-J^2^	6.76 ± 1.75	7.4 ± 2.07	0.96
*P*-value (C × ST-J)		0.99	0.99	
RBC	C	7.58 ± 0.38	7.18 ± 0.37	0.86
	ST-J	7.96 ± 1.03	7.88 ± 0.93	0.99
*P*-value (C × ST-J)		0.90	0.41	
Hb	C	129.25 ± 5.82	**121.75 ± 6.14**	0.69
	ST-J	138.5 ± 12.35	**135.57 ± 9.82**	0.99
*P*-value (C × ST-J)		0.48	**0.02**	
HCT	C	0.39 ± 0.02	0.366 ± 0.016	0.65
	ST-J	0.41 ± 0.04	0.407 ± 0.03	0.99
*P*-value (C × ST-J)		0.86	0.17	
MCV	C	51.5 ± 2.2	51.38 ± 2.26	0.99
	ST-J	51.5 ± 4.17	51.57 ± 3.86	1.0
*P*-value (C × ST-J)		1.0	0.99	
MHC	C	17.25 ± 0.70	17 ± 1.07	0.99
	ST-J	17.63 ± 1.30	17.29 ± 1.38	0.98
*P*-value (C × ST-J)		0.97	0.99	
MCHC	C	333.38 ± 9.52	333.13 ± 7.06	0.99
	ST-J	339.63 ± 7.87	335.42 ± 6.39	0.89
*P*-value (C × ST-J)		0.59	0.99	
RDW	C	17.94 ± 0.55	17.54 ± 0.38	0.99
	ST-J	17.92 ± 0.98	17.54 ± 0.79	1.0
*P*-value (C × ST-J)		1.0	1.0	
Platelets	C	130.13 ± 29.84	112 ± 26.14	0.73
	ST-J	101.5 ± 21.76	98.85 ± 20.31	0.99
*P*-value (C × ST-J)		0.25	0.92	
MPV	C	6.63 ± 0.65	6.4 ± 0.45	0.95
	ST-J	6.92 ± 0.51	6.61 ± 0.22	0.85
*P*-value (C × ST-J)		0.85	0.9	
T.S. protein	C	68.75 ± 2.71	66.88 ± 2.75	0.88
	ST-J	71.63 ± 4.17	67.71 ± 2.75	0.26
*P*-value (C × ST-J)		0.55	0.97	
Seg Neutrophils	C	3.39 ± 0.43	4.42 ± 0.79	0.44
	ST-J	3.89 ± 1.45	4.45 ± 1.69	0.91
*P*-value (C × ST-J)		0.94	0.99	
Lymphocytes	C	2.86 ± 1.02	2.75 ± 1.19	0.99
	ST-J	2.60 ± 0.79	2.62 ± 0.64	0.99
*P*-value (C × ST-J)		0.99	0.99	
Monocytes	C	0.15 ± 0.12	0.15 ± 0.08	1
	ST-J	0.18 ± 0.08	0.28 ± 0.31	0.82
*P*-value (C × ST-J)		0.99	0.73	
Eosinophils	C	0.15 ± 0.13	0.07 ± 0.01	0.99
	ST-J	0.09 ± 0.06	0.09 ± 0.08	0.99
*P*-value (C × ST-J)		0.99	0.99	
Basophils	C	0.53 ± 0.66	0.03 ± 0.04	0.25
	ST-J	0.05 ± 0.01	0.01 ± 0.01	0.99
*P*-value (C × ST-J)		0.25	0.99	
Rouleux	C	1.17 ± 0.41	0.94 ± 0.18	0.91
	ST-J	1.5 ± 0.54	1.43 ± 0.54	0.99
*P*-value (C × ST-J)		0.72	0.22	

^1^C—control horses.

^2^ST-J—horses receiving Structure-JOINT.

Values in bold are significantly different from each other (p<0.05).

#### Biochemistry profile.

Biochemistry profile data for days 0 and 30 are provided in Table 6.

**Table 6.** Biochemistry profile outcome measures

Outcome measure	Dietary treatment	Day 0	Day 30	*P*-value (Day 0 × Day 30)
Calcium	C	2.91 ± 0.09	2.94 ± 0.12	0.99
	ST-J	2.89 ± 0.08	2.91 ± 0.11	0.98
*P*-value (C × ST-J)		0.99	0.98	
Phosphorus	C	1.03 ± 0.28	0.99 ± 0.23	0.99
	ST-J	0.88 ± 0.05	0.96 ± 0.16	0.98
*P*-value (C × ST-J)		0.74	0.99	
Magnesium	C	0.7 ± 0.08	0.76 ± 0.09	0.34
	ST-J	0.74 ± 0.05	0.8 ± 0.001	0.38
*P*-value (C × ST-J)		0.82	0.84	
Sodium	C	137.62 ± 1.77	136.13 ± 1.64	0.62
	ST-J	136.25 ± 2.43	137.71 ± 1.88	0.67
*P*-value (C × ST-J)		0.70	0.59	
Potassium	C	4.625 ± 0.40	4.75 ± 0.83	0.99
	ST-J	4.58 ± 0.41	4.8 ± 0.55	0.95
*P*-value (C × ST-J)		0.99	0.98	
Chloride	C	101.75 ± 1.04	100.63 ± 2.72	0.99
	ST-J	112.7 ± 36.5	101.0 ± 2.52	0.99
*P*-value (C × ST-J)		0.69	0.99	
Carbon dioxide	C	27.25 ± 1.67	27.88 ± 2.47	0.99
	ST-J	27.0 ± 2.39	29.71 ± 2.43	0.14
*P*-value (C × ST-J)		0.99	0.54	
Anion gap	C	13.25 ± 1.49	12.5 ± 2.27	0.95
	ST-J	13.63 ± 1.59	11.85 ± 1.86	0.37
*P*-value (C × ST-J)		0.99	0.97	
NaK ratio	C	29.88 ± 2.99	29.5 ± 4.28	0.99
	ST-J	30.0 ± 2.73	29.14 ± 3.18	0.99
*P*-value (C × ST-J)		0.99	0.99	
Total protein	C	61.75 ± 4.03	62.13 ± 3.72	0.99
	ST-J	62.87 ± 4.12	62.0 ± 2.45	0.99
*P*-value (C × ST-J)		0.99	0.99	
Albumin	C	32.00 ± 2.51	32.25 ± 2.05	0.99
	ST-J	32.0 ± 2.13	32.43 ± 1.90	0.99
*P*-value (C × ST-J)		1.0	0.99	
Globulin	C	29.75 ± 4.49	29.88 ± 3.44	0.99
	ST-J	30.89 ± 4.01	29.57 ± 2.44	0.97
*P*-value (C × ST-J)		0.98	0.99	
A:G Ratio	C	1.10 ± 0.24	1.09 ± 0.17	0.99
	ST-J	1.05 ± 0.15	1.10 ± 0.12	0.98
*P*-value (C × ST-J)		0.98	0.99	
Urea	C	5.35 ± 1.17	5.39 ± 0.65	0.99
	ST-J	6.13 ± 0.87	5.83 ± 0.75	0.98
*P*-value (C × ST-J)		0.48	0.92	
Creatinine	C	107.5 ± 64.29	117.38 ± 60.46	0.99
	ST-J	100.5 ± 20.39	106.71 ± 13.97	0.99
*P*-value (C × ST-J)		0.99	0.99	
Glucose	C	5.21 ± 0.24	5.06 ± 0.24	0.99
	ST-J	5.88 ± 0.89	5.16 ± 0.42	0.06
*P*-value (C × ST-J)		0.09	0.99	
Cholesterol	C	1.81 ± 0.25	1.73 ± 0.30	0.99
	ST-J	1.93 ± 0.34	1.86 ± 0.34	0.99
*P*-value (C × ST-J)		0.97	0.95	
Total bilirubin	C	18.75 ± 3.24	**16.38 ± 2.07**	0.95
	ST-J	25.0 ± 7.05	**24.85 ± 9.83**	1.0
*P*-value (C × ST-J)		0.23	**0.05**	
Conjugated bilirubin	C	5.13 ± 1.36	5.25 ± 1.16	0.99
	ST-J	5.63 ± 1.69	6.0 ± 0.82	0.99
*P*-value (C × ST-J)		0.97	0.88	
Free bilirubin	C	13.63 ± 2.50	**11.13 ± 2.1**	0.93
	ST-J	19.38 ± 7.54	**18.86 ± 10.04**	0.99
*P*-value (C × ST-J)		0.29	**0.04**	
Alkaline phosphatase	C	121.63 ± 40.11	129.13 ± 34.12	0.99
	ST-J	98.38 ± 27.25	113.0 ± 33.57	0.97
*P*-value (C × ST-J)		0.79	0.95	
GGT	C	9.25 ± 4.97	11.25 ± 3.15	0.98
	ST-J	11.13 ± 9.54	10.43 ± 2.29	0.99
*P*-value (C × ST-J)		0.98	0.99	
AST	C	294.50 ± 57.43	307.50 ± 54.87	0.99
	ST-J	272.0 ± 25.85	267.57 ± 29.71	0.99
*P*-value (C × ST-J)		0.95	0.66	
CK	C	365.13 ± 123.49	330.00 ± 136.08	0.98
	ST-J	247 ± 67.81	291.86 ± 80.13	0.97
*P*-value (C × ST-J)		0.32	0.98	
GLDH	C	6.25 ± 3.54	4.63 ± 2.62	0.77
	ST-J	5.88 ± 2.10	3.48 ± 0.53	0.98
*P*-value (C × ST-J)		0.93	0.99	
Calculated osmolality	C	275.13 ± 3.18	272.5 ± 3.82	0.99
	ST-J	273.85 ± 4.85	276.14 ± 4.37	0.87
*P*-value (C × ST-J)		0.98	0.49	
Serum amyloid A	C	1.60 ± 1.27	0.613 ± 0.704	0.86
	ST-J	0.86 ± 0.09	0.31 ± 0.28	0.99
*P*-value (C × ST-J)		0.99	0.99	

Values in bold are significantly different from each other (p<0.05).

On day 30, ST-J horses had higher total bilirubin (*P* = 0.05), free bilirubin (*P* = 0.04) and hemoglobin (*P* = 0.02) than CON horses on the same day. In addition, glucose tended (*P* = 0.06) to be lower in ST-J horses than CON horses on day 30.

## DISCUSSION

The purpose of this experiment was to determine the effects of feeding a dietary supplement ST-J for 30 d in an equine model of mild articular inflammation. The main findings were that supplementation with ST-J for 30 d significantly reduced joint circumference and increased synovial fluid PGE_2_ compared to baseline in the same group of horses. Furthermore, on day 30 NO and RvD1 were increased in ST-j horses compared to control horses. There was also a significant increase in plasma hemoglobin, free and total bilirubin, and a decrease in plasma glucose.

The intra-articular model utilized in the current experiment was designed to challenge articular tissues to launch a transient inflammatory response that is representative of what can be expected following a single high-intensity exercise bout; i.e., produces physiological evidence of articular inflammation whilst minimizing clinical impact on the research horses. Use of intra-articular reIL-1 β to induce synovitis is not novel, but most literature reports an intercarpal dose of 100 ng (Pearson et al., 2009; Ross et al., 2012; Ross-Jones et al., 2016). This dose is associated with increased clinical lameness of approximately 3 lameness points (AAEP Lameness Scale) at PIH 4 to 8 (Ross et al., 2012). One previous study reports use of a 75 ng dose to transiently induce synovial fluid biomarkers of articular inflammation (Colbath et al., 2018) suggesting that the 100 ng challenge may be unnecessarily high. Thus, we opted for a 75 ng dose, with the intention to limit as much as possible the clinical consequences of the injection whilst still promoting a pro-inflammatory physiological response. Unlike the previous Colbath study, we did not see a significant increase in lameness at any time point. Our result may have been confounded by the practical limitation that several of our research horses (2 in ST-J group; 6 in CON group) started the study with a positive lameness exam, sometimes in a limb that was not challenged with the reIL-1 β injection. Although statistically the ST-J group had lower lameness score that CON group, this is likely an artifact of randomization which by chance placed more horses with baseline lameness in the CON group than the ST-J group. Therefore, additional studies are needed to determine the effects of ST-J on lameness.

The increase in rectal temperature at PIH 6 of approximately 0.5 °C in the current study contradicts others who report no effect of 75 ng reIL-1 β on rectal temperature (Colbath et al., 2018). The reason for this discrepancy is unclear but may be associated with different ambient temperatures between these two experiments. Rectal temperature is sensitive to ambient temperature, humidity, and ventilation (Giannetto et al., 2022); none of these variables were reported (Colbath et al., 2018) or recorded (current study) and should be controlled for in future studies that utilize 75 ng as an intra-articular challenge dose.

Previously, an intra-articular challenge dose of 75 ng was associated with an increase in joint circumference at PIH 72 (Colbath et al., 2018), which is in contrast with our findings of no effect of reIL-1 β at any time point. The two studies utilized the same number of horses (*n* = 8), with the Colbath group taking more frequent synovial fluid samples (5 samples) in the first 72 h compared with the current study (4 samples), which may have contributed to the differing results on joint circumference. While reIL-1 β did not influence joint circumference, dietary treatment had a significant effect with ST-J horses showing a significant reduction in joint circumference between day 0 and day 30. While this may indicate a stabilizing effect of the ST-J on joint effusion, enthusiasm for this result should be tempered by the fact that the ST-J group began the study with higher joint circumference than the CON group. Therefore, it is possible that this elevated effusion may have resolved spontaneously. Future studies are needed to clarify the effect of ST-J on articular effusion.

Consistent with previous research (Colbath et al., 2018), 75 ng of intra-articular reIL-1 β in the current study resulted in significant increase in synovial fluid PGE_2_ at PIH 6. While persistently elevated PGE_2_ is associated with increased articular breakdown and joint disease (Schulze-Tanzil et al., 2011; Liu et al., 2016; Jasiński et al., 2024), transiently elevated PGE_2_ is associated with increased tissue turnover and tissue adaptation to exercise stress (Trappe et al., 2013), enhancing anaerobic capacity and reducing the inflammatory response to training (Roberts et al., 2007; Capó et al., 2016), facilitating bone strength and regeneration (Ho et al., 2017), increasing angiogenic growth factors in skeletal muscle (Benoit et al., 1999), increasing exercise-induced arteriolar vasodilation in muscle (Wilson and Kapoor, 1993), and upregulating production of hormones implicated in increased exercise tolerance (Di Luigi et al., 2001). The latter effect is attributed to the algesic properties of PGE_2_, which are the target of pain-relieving drugs such as phenylbutazone (“bute”). But far from being inherently negative, PGE_2_ responses to stress are essential to an organized adaptation of tissue and resistance to damage from subsequent exposures to physiologically stressful events. The augmentation of PGE_2_ observed in ST-J horses by day 30 in the current study was not associated with an increase in lameness, perhaps because of the concurrent increase in baseline levels of RvD1. RvD1 is an elongation product of docosohexaenoic acid (DHA) with potent antiinflammatory properties (Sun et al., 2007). RvD1 has an inverse relationship with exercise-induced PGE_2_ and appears to either preempt or abbreviate its persistence in blood (Pearson et al., 2020), and the combination of exercise-induced increase in PGE_2_ in the presence of RvD1 has antiinflammatory effects in the context of exercise training (Capó et al., 2016). RvD1 and PGE_2_ are derived from polyunsaturated omega-3 (DHA) and omega-6 (linoleic acid) fatty acids, respectively. The increase in RvD1 and PGE_2_ observed in the ST-J horses in the current study may have resulted from increased availability of substrate for their formation provided by the ST-J supplement. These data provide evidence of a role for ST-J in facilitating adaptation of articular tissues to exercise-induced stress. Given the dualistic roles of PGE_2_ in tissue adaptation to exercise stress vs. those in inflammatory disease, we cannot exclude the possibility that an ST-J-mediated increase in PGE_2_ could contribute to an increase in articular damage. Our study does not provide evidence for this, because synovial fluid concentrations prior to and following an IL-1 injection were consistent with those seen at 8 h postexercise in healthy horses (Frisbie et al., 2008). And we did not see a significant increase in GAG, which may have indicated an increase in cartilage loss. However, additional studies of longer duration, which use an exercise model of physiological stress, are needed to further evaluate long-term implications of enhanced PGE_2_ response.

Like PGE_2_, NO is also a complex mediator of tissue adaptation to inflammatory and exercise stress. In the context of joint disease, NO has dualistic catabolic and anabolic roles, the relative balance of which depends on the magnitude and persistence of NO elevation (reviewed in Ahmad et al., 2020). NO is a potent angiogenic factor and vasodilator and reduces peripheral resistance during exercise (Benoit et al., 1999). It is critical to exercise and training (Gilligan et al., 1994; Arefirad et al., 2022), and it functions to promote adaptation of tissues during conditions where oxygen supply and demand are unbalanced as in high-intensity exercise (Umbrello et al., 2013; Premont et al., 2020). NO is carried on Hb molecules and dilates the microvasculature to increase local blood flow and oxygen delivery to tissue (Premont et al., 2020). Supplementation of substances that enhance NO production is associated with improved exercise performance and training (Kiani et al., 2022; Jędrejko et al., 2024) through mechanisms that include improving blood flow during physical exertion, reducing oxygen demand, and improving mitochondrial function (Sebastiá-Rico et al., 2023). Thus, the increase in NO observed in ST-J horses compared to CON horses in the current study provides further support for a hypothesis of improving adaptation of tissues to exercise stress.

The mechanism by which ST-J increases NO is not known, but the increase in NO may have contributed to the higher Hb concentration observed in ST-J horses compared with CON horses on day 30. Hb is a protein within erythrocytes (and a few other cells with affinity for oxygen) that binds oxygen, carbon dioxide, and NO (Ramsook et al., 2023). Hemoglobin in erythrocytes undergoes autoxidation at a rate of approximately 3% every 24 h (Kuhn et al., 2017). This oxidation rate is influenced by many factors, but a key one is availability of NO (Angelo et al., 2006). Hb affinity for NO increases during increased cellular production of NO (as in during exercise), which can prolong a reduced state in the Hb molecule (Angelo et al., 2006). Indeed, the bioactivity of NO is also prolonged when it is bound to Hb, which may have contributed to the increased NO observed in the ST-J horses. Interestingly, there is evidence that oxidation of Hb can be tempered by provision of antioxidant supplements such as linoleic acid derived from fish oil (Walczewska et al., 2010). Regardless of the physiological strategies by which Hb and NO communicate, the concurrent increase in both these compounds following 30 d of ST-J supplementation may indicate evidence for a beneficial effect of the supplement on exercise performance. Similar to findings for PGE_2_, the authors acknowledge the possibility that ST-J-mediated augmentation of NO could have negative implications for horses at risk for development of inflammatory diseases, such as arthritis, due to the role of NO in propagation of this condition (Ahmad et al., 2020). Future studies should investigate this possibility by following ST-J-supplemented exercising horses under an intentional lens of articular health.

The current study showed no significant effects of ST-J on dynamics of cartilage turnover, with no significant differences from baseline or compared to CON horses in GAG release (i.e., proteoglycan breakdown and release into synovial fluid) or CS846 [an epitope of chondroitin sulfate which is positively correlated with proteoglycan synthesis (Knych et al., 2023)]. While there was a tendency to lower CS846 in ST-J horses compared with CON, the ST-J group started with lower CS846 before supplementation, and this numerical difference persisted almost identically through day 30. The lack of effect of 75 ng reIL-1 β in either group of horses suggests that the chemical challenge may not have been sufficient to provoke increased cartilage turnover at the time points measured within our 72 h postinjection period. Others have shown a decrease in activity of ADAMTS-4 in horses following 90 d of a long-chain fatty acid supplement (Ross-Jones et., 2016), which indicates that effects of ST-J on cartilage metabolism may require a longer supplementation period than used in the current study. Thus, further studies on ST-J with the aim to increase understanding of its effects on cartilage metabolism should use a higher (100 ng) dose of reIL-1 β as the intra-articular challenge and ST-J should be fed for a minimum of 90 d.

Other notable effects of ST-J in the current study included a decrease in blood glucose levels. Others have also reported this effect of PUFA supplementation in humans (Mone et al., 2022), rodents (Wang et al., 2021) and horses during exercise recovery (O’Connor et al., 2004). This effect is likely due, at least in part, to a PUFA-mediated increase in insulin sensitivity and modulation of gluconeogenesis (Li et al., 2019). Improvement in glucose dynamics via improved insulin sensitivity during exercise recovery has potentially important implications for glycogen resynthesis. Glycogen resynthesis in horses is protracted compared with humans due in part to sluggish postexercise insulin (Pratt et al., 2007) and limited availability of lipids for energy production (Hyyppä et al., 1997). The suppressive effect of ST-J on blood glucose and its impact on postexercise glycogen resynthesis should be explored in future studies.

Total and free bilirubin was higher in ST-J horses compared with CON horses on day 30. This likely also occurred as an artifact of randomization as ST-J horses were numerically higher than CON horses on day 0. While this difference reached statistical significance on day 30, total and free bilirubin concentrations were numerically lower in both groups. And these variables always remained well within normal reference intervals for all horses.

Data from this study must be interpreted within the context of its limitations. The number of research horses was quite low, which is a common limitation of equine studies. We blocked groups according to age and body mass, but randomization still resulted in baseline values, which were at times statistically and/or physiologically relevant, and it is possible that these randomization artifacts may have affected outcome measures. In particular, baseline lameness differences presented a functional disparity between groups that should be avoided in future studies. Another limitation is that our research horses were fed a forage diet that is not expected to provide a high Ω6 to Ω3 ratio, which may have influenced our results. Future studies with ST-J should include analysis of fatty acid composition of blood and/or tissues. It is also possible that the results of this study would have differed if the ST-J supplement had been fed for a longer duration than the current 30 d. Additionally, the reIL-1 β model employed in the current study, while producing some outcome measures that are similar to those produced by exercise, cannot account for the systemic stress of an exercise bout. Therefore, it is recommended that future studies explore the effects of ST-J in exercising horses.

## CONCLUSION

It is concluded that supplementation of healthy horses with ST-J for 30 d improved physiological parameters with relevance to exercise performance and tissue adaptation to training. Further studies on this supplement to differentiate beneficial and nonbeneficial inflammatory effects are warranted.

## Abbreviations

A: albumin
AHL: Animal Health Laboratory
AST: aspartate aminotransferase
CON: control
CS846: chondroitin sulfate epitope 846
DHA: docosahexaenoic acid
G: globulin
GAG: glycosaminoglycan
GGT: gamma glutamyl transferase
GLDH: glutamate dehydrogenase
HCT: hematocrit
Hb: hemoglobin
IL-1: recombinant equine interleukin 1β
K: potassium
MCHC: mean corpuscular hemoglobin concentration
MCV: mean corpuscular volume
MHC: mean hemoglobin content
MPV: mean plasma volume
Na: sodium
NO: nitric oxide
PGE2: prostaglandin E2
PIH: postinjection hour
PUFA: polyunsaturated fatty acid
RBC: red blood cell count
RDW: red cell distribution width
RvD1: resolvin D1
Seg neutrophils: segmented neutrophils
ST-J: STRUCTURE-Joint
T.S. protein: total solid protein
WBC: white blood cell count
